# Incorporating Patient-Reported Outcome Measures and Patient-Reported Experience Measures in Addiction Treatment Services in Belgium: Naturalistic, Longitudinal, Multicenter Cohort Study

**DOI:** 10.2196/65686

**Published:** 2025-04-30

**Authors:** Amine Zerrouk, Charlotte Migchels, Clara De Ruysscher, Kim Fernandez, Jerome Antoine, Florian De Meyer, Frieda Matthys, Wim van den Brink, Cleo Lina Crunelle, Wouter Vanderplasschen

**Affiliations:** 1 Department of Special Needs Education Ghent University (UGent) Ghent Belgium; 2 Department of Psychiatry Universitair Ziekenhuis Brussel (UZ Brussel) Vrije Universiteit Brussel (VUB) Brussels Belgium; 3 Department of Epidemiology and Public Health Sciensano Brussels Belgium; 4 Department of Psychiatry Amsterdam University Medical Centers University of Amsterdam Amsterdam The Netherlands

**Keywords:** patient-reported outcome measures, patient-reported experience measures, substance use disorder, recovery, ICHOM, International Consortium for Health Outcomes Measurement, addiction, PROMs, PREMs, SUD, treatment, protocol, substance use, inpatient, services, perspectives, treatment outcome

## Abstract

**Background:**

Traditionally, treatment outcomes of service users with a substance use disorder (SUD) are measured using objective and provider-reported indicators. In recent years, there has been a shift toward incorporating patient-reported outcome measures (PROMs) and patient-reported experience measures (PREMs) to capture service users’ perspectives on treatment outcomes and experiences.

**Objective:**

The OMER-BE (Outcome Measurement and Evaluation as a Routine Practice in Alcohol and Other Drug Services in Belgium) study evaluates the acceptability and feasibility of PROMs and PREMs in different SUD treatment services, using the recently developed International Consortium for Health Outcomes Measurement Standard Set for Addictions. This paper presents the design and baseline characteristics of the study, indicators of attrition at 45-day follow-up, and the feasibility of the implementation of PROMs and PREMs in residential and outpatient services.

**Methods:**

A convenience sample of 189 treatment-seeking individuals with SUD from different inpatient (therapeutic communities and psychiatric centers) and outpatient treatment services was followed for six months. Sociodemographic characteristics; clinical factors; and PROMs including recovery strengths, quality of life, and global health were assessed at baseline and within 3 weeks after starting treatment. Additionally, PROMs and PREMs were measured 45, 90, and 180 days later. Comparisons were made between treatment modalities, and indicators of attrition at the 45-day follow-up were assessed using ANOVA and chi-square tests.

**Results:**

Baseline differences were observed between the three treatment modalities regarding education, SUD treatment history, primary substance, and Attention-Deficit/Hyperactivity Disorder Self-Report scores. Overall, patients in psychiatric treatment centers had a higher education level and less polysubstance use, while outpatients had fewer previous SUD treatments but received relatively more often opioid agonist treatment. Inpatients reported more attention-deficit/hyperactivity disorder symptoms and higher SUD severity than outpatients. Additionally, recovery strength scores were significantly lower in the outpatient group compared to the other groups, particularly in the subdomains of “Substance Use,” “Self-care,” and “Outlook on Life.” At the 45-day follow-up assessment, the attrition rate was 36.6%. Comparisons between participants who completed the 45-day follow-up and those who dropped out revealed that completers were significantly older, had a higher level of education, were more likely to live alone, and were more likely to have a mother born in Belgium. They also had higher average scores on the “Material Resources” domain of the Substance Use Recovery Evaluator, which includes questions about stable housing, a steady income, and effective financial management.

**Conclusions:**

Evaluating PROMs and PREMs appears to be feasible in a diverse group of treatment-seeking patients with SUD in Belgium. However, challenges remain for structural implementation in practice, especially in outpatient services. Routine monitoring of PROMs and PREMs has the potential to empower patients, service providers, and policy makers by providing a comprehensive understanding of service users’ needs and treatment effectiveness.

## Introduction

Alcohol and other substance use disorders (SUDs) are linked to a range of adverse psychological, physical, and social consequences. Their chronic, relapsing nature and related judicial, housing, and relational problems impact individuals with SUDs, as well as their environment and the broader community [1-4]. SUDs have a significant and growing impact on global morbidity and mortality [5-7]. Worldwide, harmful alcohol use causes 3 million deaths annually, representing 5.3% of all deaths. Alcohol use accounts for 5.1% of the global burden of disease [8]. Furthermore, an estimated 60,000 years of life lost were attributed to drug use in Europe in 2019 [5].

Treatment cohort studies conducted in the United States, Australia, and various European countries have shown the benefits of engaging in SUD treatment, generally resulting in increased abstinence rates, improved social integration, and reduced psychopathology [9-12]. Various SUD treatment modalities, however, seem to impact treatment outcomes in different ways. Stahler et al [13], for example, found that people with problematic opioid use benefitted more from residential treatment than individuals with alcohol as their primary substance. Conversely, people with problematic cannabis use were less likely to benefit from residential treatment compared to people with alcohol as their primary substance. Several studies found higher treatment completion rates in residential programs compared to outpatient settings [13,14]. Treatment engagement and retention have been consistently associated with positive outcomes, irrespective of the treatment modality [15-17]. Individuals who stay in treatment for a longer period of time are more likely to remain abstinent, experience fewer relapses and readmissions, engage less in criminal activity, and show greater improvements in general health measures [13,14,18]. Despite evidence for the effectiveness of SUD treatment, significant challenges such as high dropout and relapse rates persist and warrant further research and innovative approaches [16].

SUD treatment outcomes are typically measured using objective (eg, abstinence and rearrest or reincarceration) or provider-reported indicators (eg, treatment completion or compliance and absence of symptoms). Increasing emphasis on service users’ perspectives in measuring treatment outcomes and experiences has recently led to the introduction of patient-reported outcome measures (PROMs) and patient-reported experience measures (PREMs) in SUD and other mental health services [19-22]. PROMs refer to individual, subjective treatment outcomes, including information about psychological well-being, quality of life, symptomatology, and physical functioning. PREMs provide information on how individuals experience health care and measure practical aspects of care, such as coordination, continuity and accessibility of care, and quality of patient-provider relationships [23]. International organizations, such as the International Consortium for Health Outcomes Measurement (ICHOM) [24], promote the implementation of patient-reported measures for routine assessment of health outcomes and experiences in all health care areas, including primary care, psychotherapy, and SUD services [21]. The use of PROMs and PREMs is advised to systematically monitor and improve the delivery of effective, patient-centered care and shared decision-making, which is not a standard practice in SUD and other mental health services [20,21,25-30]. Additionally, adopting standardized outcome measures opens opportunities to compare performances between treatment services within regions, countries, and even globally. This evolution will facilitate knowledge sharing among practitioners and provide policy makers with the tools and evidence needed to improve the quality and effectiveness of care [24]. The systematic use of PREMs is also likely to advance the field toward more personalized and effective support since it provides a direct evaluation of the accessibility, continuity, and coordination of care by service users [21,23]. Yet, although PROMs and PREMs show clear promise in improving the quality of SUD treatment, there is a lack of research on (the implementation of) these measures in clinical practice within SUD treatment settings [21]. The OMER-BE (Outcome Measurement and Evaluation as a Routine Practice in Alcohol and Other Drug Services in Belgium) study (2022-2025) aims to introduce and integrate the use of PROMs and PREMs in different SUD treatment modalities and to evaluate and enhance the quality of care provided in SUD treatment services in Belgium. The primary objective of the OMER-BE study is to assess the routine measurement of PROMs and PREMs in SUD treatment services, based on the recently developed and internationally validated ICHOM Standard Set for Addictions (ICHOM SSA) [24]. Therefore, we translated and validated the ICHOM SSA into Dutch and French, enhancing its applicability in Belgium and other Dutch- and French-speaking countries. By incorporating PROMs and PREMs in routine assessments, SUD treatment services are provided with information to better align treatment approaches with service user needs. The secondary objective of the OMER-BE study is to measure and compare various recovery indicators and treatment experiences between different treatment modalities and assess their evolution during and after treatment. In this paper, we describe the design and baseline characteristics of the study. In order to enhance study adherence and routine implementation of PROMs and PREMs, we also examined indicators of attrition.

## Methods

### Study Setting

The OMER-BE study was a naturalistic, longitudinal, multicenter cohort study, in which 189 individuals with SUDs were followed up over a 6-month period in various residential and outpatient treatment modalities in the Dutch- and French-speaking regions of Belgium. Data collection for the study started in July 2022. Participants were recruited from 8 residential services (4 psychiatric treatment centers [PCs] and 4 therapeutic communities [TCs]) and 9 outpatient treatment services.

Specialized wards of PCs offer long-term (3 to 6 months) residential care that provides intensive medical and psychological support, addressing SUDs and in some cases co-occurring mental health disorders. Treatment consists of group counseling, psychoeducation, individual psychotherapy, and occupational activities.

TCs for addictions have a long history and were set up for individuals with SUDs, complementing traditional mental health care services that were traditionally not open to persons with drug problems [31]. In a TC, individuals with SUDs live together in a structured environment, typically for a period of 6 to 12 months, aiming for positive changes that lead to a drug-free life in society. The TC approach centers around the concept of “community as a method,” thus highlighting the influential role of peers and the power of mutual support in fostering recovery [32,33].

Outpatient treatment services provide more autonomy to service users and offer various nonresidential care options consisting of drug-free counseling interventions and harm-reduction approaches like opioid agonist therapy (OAT) and needle exchange programs. OAT refers to the use of opioid replacement medication such as methadone or buprenorphine to help manage withdrawal symptoms and reduce craving, often combined with some form of counseling and social support. Drug-free counseling focuses on psychosocial interventions, such as motivational interviewing and cognitive behavioral therapy, aimed at helping individuals develop coping strategies and support systems to become or remain abstinent.

### Study Sample

The OMER-BE study aims to follow up a naturalistic cohort of service users with SUDs as they start a new treatment episode in a selected number of SUD treatment services, focusing on individuals with a primary alcohol or primary (illicit) drug problem. Eligibility criteria were (1) having a documented SUD (eg, a *Diagnostic and Statistical Manual of Mental Disorders* diagnosis of a SUD or previous treatment for a SUD), (2) being at least 18 years old, (3) being able to communicate in Dutch or French, and (4) having started treatment no longer than 21 days ago. First-time users, as well as returning service users, were considered eligible for this cohort study, as long as they started a new treatment episode during the recruitment period [34].

Upon treatment entry, service users were informed about the aims and design of the OMER-BE study through posters, leaflets, and staff members of the selected treatment facilities. During the initial meeting with the researcher, participants were informed extensively about the (follow-up) study and the implications of study participation and were asked for written informed consent to participate [34].

### Study Procedure

#### Baseline Assessment

Sociodemographic and clinical factors (Textbox 1) were assessed at baseline, followed by the assessment of PROMs (Textbox 1). Data were collected through self-report using a tablet provided by one of the researchers, who were available throughout the assessment to address any questions participants may have. Administering the set of baseline variables and PROMs took between 20 and 45 minutes. After completion of the baseline assessment, participants received a voucher of €10 (US $10.46) as remuneration.

**Overview of included instruments in the OMER-BE (Outcome Measurement and Evaluation as a Routine Practice in Alcohol and Other Drug Services in Belgium) outcome measurement tool.**

**Sociodemographic factors**

- Year of birth, sex, highest level of education completed, housing status, ethnicity

**Clinical factors**

- Treatment history for substance use disorders
- Primary Care Posttraumatic Stress Disorder Screen for Diagnostic and Statistical Manual of Mental Disorders (PC-PTSD-5)
- Depression, Anxiety, and Stress Scale (DASS-21)
- Adult Attention-Deficit/Hyperactivity Disorder Self-Report Scale (ASRS-v1.1)

**Patient-reported outcome measures**

- Patient-Reported Outcomes Measurement Information System (PROMIS) Short Form v1.0—Alcohol Use 7a (PROMIS-Alcohol)
- PROMIS SF v1.0—Severity of Substance Use 7a (PROMIS-Substance)
- Heaviness of Smoking Index (HSI)
- National Health Service Treatment Outcomes Profile for Substance Misuse—section 1 (TOP-S1)
- PROMIS Scale v1.2—Global Health (PROMIS-GH-10)
- Substance Use Recovery Evaluator (SURE)
- Brief version of the World Health Organization Quality of Life Scale (WHOQoL-BREF)

**Patient-reported experience measures**

- Patient-Reported Experience Measure for Addiction Treatment (PREMAT)

#### Follow-up Assessments

**Figure 1:**
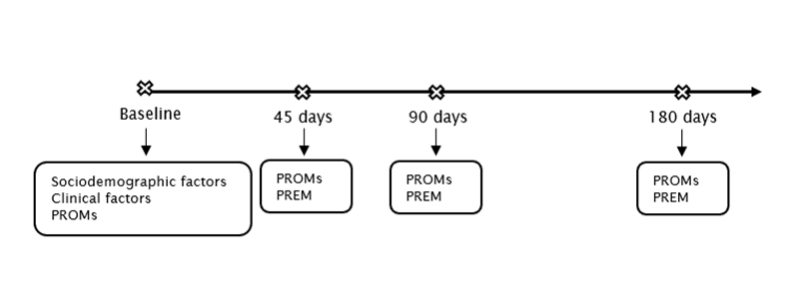
Overview of the data collection process. PREM: patient-reported experience measure; PROM: patient-reported outcome measure.

Study participants are contacted again 45, 90, and 180 days after the baseline assessment (Figure 1). We use a time window of four weeks for the 45- and 90-day follow-up and five weeks for the 180-day follow-up assessment to collect and complete the questionnaires. Researchers contacted all study participants either via email, SMS text messages, directly by phone, or through the treatment setting where they were enrolled in the study. Participants can complete the online survey via a personalized link provided by email or during a face-to-face or telephone interview, depending on their preference. The follow-up assessments include a measurement of both PROM and PREM variables (Textbox 1), which are supposed to take 20 to 30 minutes to complete. Participants receive a €10 (US $10.46) voucher for each completed follow-up assessment.

As the OMER-BE project is a naturalistic study, some participants will no longer be in treatment at the 45-, 90- or 180-day follow-up. Consequently, the PREM instrument is only administered when service users are still in treatment and at the first follow-up moment after leaving treatment. For example, if a participant stops treatment after 50 days, the PREM questionnaire will be administered at the 45- and 90-day follow-up moments.

### Instruments

#### Overview

The baseline and follow-up assessments (Textbox 1) are largely based on the ICHOM SSA [24], a set of brief, existing, validated questionnaires to measure and monitor treatment outcomes routinely in SUD services that were developed by an international panel of SUD specialists. The ICHOM SSA focuses on patient-centered outcome indicators and provides an internationally agreed-upon method for measuring a variety of outcome domains. The tool offers potential for routine use since it is relatively short and has been specifically developed for and validated in the population of SUD service users. It can be easily administered and is applicable in a wide range of treatment settings [24]. To facilitate its application in Belgium, nontranslated questionnaires were translated into French and Dutch using forward or backward translation, following guidelines provided by Tsang et al [35], and subsequently validated. Compared to the ICHOM SSA procedure, we added a 45-day follow-up assessment, which allowed us to have an additional measurement point, keep participants more engaged in the study, and reduce attrition.

The OMER-BE measurement tool consists of three sections (Textbox 1): (1) sociodemographic and clinical factors, (2) PROMs, and (3) PREM.

#### Sociodemographic and Clinical Factors

##### Overview

The first section of the tool includes sociodemographic and clinical factors that may influence treatment outcomes.

The following sociodemographic variables were assessed: age, sex, education level, current living situation, country of birth of the participants, and country of birth of their parents.

Clinical factors include questions regarding SUD treatment history and three validated and widely used screening instruments to assess common comorbid psychiatric disorders (trauma, depression or anxiety, and attention-deficit/hyperactivity disorder [ADHD]) that are likely to affect treatment outcomes. These clinical factors were included to ensure a comprehensive understanding of how these variables influence recovery trajectories.

##### Primary Care Posttraumatic Stress Disorder Screen for Diagnostic and Statistical Manual of Mental Disorders

The Primary Care Posttraumatic Stress Disorder (PTSD) Screen [36] was developed to assess the occurrence of symptoms of PTSD over the last month. This 5-item screening tool is rated on a binary scale (No=0 and Yes=1). Higher total sum scores suggest the presence of more PTSD symptoms. The reliability of the English version has been found satisfactory in a sample of people with SUDs, as measured by a Cronbach α value of 0.73 [37].

##### Depression, Anxiety, Stress Scale

The Depression, Anxiety, and Stress Scale [38] is a commonly used instrument consisting of 21 items, divided into three subscales (depression, anxiety, and stress), each containing seven items. Each question is rated on a scale from 0=did not apply to me at all to 3=applied to me very much. Subscales and total scores are calculated by adding up the scores on the items and multiplying these by a factor of 2. In a population of Dutch-speaking SUD service users in the Netherlands, Beaufort et al [39] found the total score to be highly reliable (Cronbach α=0.91).

##### Adult ADHD Self-Report Scale

The Adult ADHD-Self-Report Scale v1.1 consists of several questions encompassing each of the 18 symptoms cited in the *Diagnostic and Statistical Manual of Mental Disorders*, *Fourth Edition* [40]. This study uses the short version, consisting of six items that have been found the most predictive of an ADHD diagnosis. These items are rated on a scale from 0=never to 4=very often. Scores equal to or higher than 2 for items 1-3 and equal to or higher than 3 on items 4-6 are indicative of a diagnosis of ADHD. A Spanish study by Blanco et al [41] showed a sensitivity of 0.88 and a specificity of 0.69 for a diagnosis of ADHD in a sample of outpatients with SUDs. In an international study among treatment-seeking in- and outpatients with an SUD, very similar results were obtained with a sensitivity of 0.83 and a specificity of 0.68 for *Diagnostic and Statistical Manual of Mental Disorders* ADHD [42].

#### PROMs

##### Overview

The second section of the tool is focused on PROMs and is based on the ICHOM SSA [24]. To extend the focus to subjective well-being beyond substance use and health outcomes, we added a widely used instrument to assess the quality of life and overall well-being (ie, brief version of the World Health Organization Quality of Life questionnaire [WHOQoL-BREF]).

##### Patient-Reported Outcomes Measurement Information System Alcohol Use Short Form 7a

The Patient-Reported Outcomes Measurement Information System (PROMIS) Alcohol Use Short Form [43] is a 7-item self-report questionnaire, derived from the 37-item PROMIS Alcohol Use item bank. This tool is designed to assess alcohol use in the last 30 days. Respondents were instructed to complete the seven questions only if they consumed alcohol in the last 30 days. Answers are rated on a 5-point scale ranging from 0=never to 4=almost always. The internal consistency of the total score has proven to be excellent in participants from the general population and a clinical sample of service users in treatment for SUDs (Cronbach α=0.95) [43].

##### PROMIS SF v1.0—Severity of Substance Use 7a

The PROMIS Severity of Substance Use Short Form [44] consists of seven items and is a shorter version of the 37-item PROMIS Severity of Substance Use item bank. This tool is designed to assess the severity of substance use in the last 30 days. Answers are rated on a 5-point scale ranging from 0=never to 4=almost always. The internal consistency of the total score was found to be excellent in participants from the general population and a clinical sample of service users in treatment for SUDs (Cronbach α=0.94) [44].

##### Heaviness of Smoking Index

The Heaviness of Smoking Index consists of two items: “How soon after waking up do you usually have your first smoke?” and a question assessing the number of cigarettes smoked each day. The first question was rated on a 4-point scale ranging from more than 60 minutes (0) to less than 5 minutes (3). The second question was replaced by an assessment of the number of cigarettes smoked in the last 30 days to maintain consistency with the assessment of the other substances. Internal consistency for the total Heaviness of Smoking Index score was found to be relatively low in a sample of male individuals with SUDs and nicotine dependence (Cronbach α=0.49) [45].

##### National Health Service Treatment Outcomes Profile for Substance Misuse—Section 1

The Treatment Outcomes Profile [46] is a multidimensional assessment instrument for monitoring outcomes in SUD treatment. This questionnaire measures four key life domains (substance use, crime, health, and social functioning). For this study, we only assessed the substance use domain and made modifications to the time frame in line with the other questionnaires. Specifically, participants were asked about their primary substances of use and the number of days and quantity consumed over the past 30 days.

##### PROMIS Scale v1.2—Global Health

The PROMIS Global Health consists of 10 items, scored on a 5-point scale. Due to overlap with other questionnaires, and in accordance with the ICHOM SSA, we only included two items of this scale relating to physical and mental health. The response options of these items are “excellent,” “very good,” “good,” “fair,” and “poor.” Its psychometric properties were evaluated using a sample of 4370 individuals from the Dutch general population. Results indicated a 2-factor structure with good internal consistency. The subscales “Global Mental Health” and “Global Physical Health” had Cronbach α of 0.83 and 0.78, respectively [47].

##### Substance Use Recovery Evaluator

The Substance Use Recovery Evaluator (SURE) [48] is a self-report questionnaire consisting of 21 items, which is completed on a 5-point scale but scored on a 3-point scale (1-3). The first two response options correspond to a score of 3, the third response option to a score of 2, and the final 2 response options to a score of 1. Response options for the first three questions are “never,” “on 1 or 2 days,” “on 3 or 4 days,” “on 5 or 6 days,” and “every day,” while for the remaining questions, response options are “all of the time,” “most of the time,” “a fair amount of the time,” “a little of the time,” and “none of the time.” Higher total sum scores suggest greater recovery strengths. The items are categorized into five subscales: “substance use,” “relationships,” “self-care,” “outlook on life,” and “material resources.” Internal consistency of the SURE total score was found to be high in a sample of current and former SUD service users (Cronbach α=0.92) [48]. Psychometric properties of the translated Dutch version of the SURE showed good internal consistency (Cronbach α=0.83) [49].

##### World Health Organization Quality of Life Scale

The WHOQoL-BREF [50] consists of 26 items and is a short version of the WHOQoL-100 questionnaire. Questions are rated on a 5-point scale (1-5) and response options, from the lowest to highest score, are “very poor/very dissatisfied/not at all/never,” “poor/dissatisfied/a little/seldom,” “neither poor nor good/neither satisfied nor dissatisfied/a moderate amount/moderately/quite often,” “good/satisfied/very much/mostly/very often,” and “very good/very satisfied/an extreme amount/extremely/completely/always.” Higher sum scores are an indication of a better quality of life. The items are grouped into four domains: “psychological health,” “physical health,” “environment” and “social relationships.” The WHOQoL-BREF has demonstrated good internal consistency with Cronbach α values for each of the domains ranging from 0.66 to 0.84 [51].

#### PREMs

##### Overview

As the objectives of the OMER-BE study also included the measurement of PREMs, the third section of the OMER-BE outcome measurement tool is not part of the ICHOM SSA. We use a newly validated PREM, the Patient-Reported Experience Measure for Addiction Treatment (PREMAT), to assess service users’ experiences regarding the treatment they received.

##### PREMAT

The PREMAT is a recently developed relatively brief (23-item) questionnaire that aims to capture the experiences of people in residential SUD treatment services [52-54]. More precisely, the following topics are addressed in the PREMAT: “Individualized support,” “Self-determination and Empowerment,” “Program structure,” “Treatment environment,” “Coordination of care,” and “Personal responsibility” [53,54]. The instrument consists of 23 statements rated on a 5-point Likert scale: 1=strongly disagree, 2=disagree, 3=neither agree nor disagree, 4=agree, and 5=strongly agree. The total score ranges from 23 to 115, with higher scores reflecting a more positive experience. Additionally, the PREMAT includes 2 open-ended questions (“How could your experience at this service have been improved?” and “What have been the best things about your experience here?”), which allows respondents to elaborate on certain aspects of the questionnaire or discuss topics that are not covered by the PREMAT items [52]. Internal consistency for the total score was found excellent in a sample of participants from specialist residential SUD treatment services in Australia (Cronbach α=0.91) [53].

### Ethical Considerations

This study was granted ethical approval from the Medical Ethics Committee of the University Hospital Brussels on May 11, 2022 (UZ Brussel; BUN: 1432022000071). The participants were informed about the confidentiality of the data they provide, and written informed consent was acquired from all participants before being included in the study. Participants received a €10 (US $10.46) voucher for each completed assessment. All data are treated confidentially and reported anonymously. This study was conducted in accordance with the Declaration of Helsinki.

### Data Analysis of Baseline Characteristics

This paper presents baseline characteristics of the study cohort and comparative analyses between participants from different treatment modalities, as well as between participants who completed the 45-day follow-up and those who did not participate in the 45-day assessment. For categorical demographic and patient characteristics, significant differences between treatment modalities were assessed using either the chi-square test or Fisher exact test when the data did not meet the assumptions required for the chi-square test. For continuous variables, we used ANOVA to assess group differences. Welch 2-tailed *t* test was used when the assumption of equal variances was not met. Post hoc comparisons between groups were made using the Tukey test when variances were equal or the Games-Howell test when the assumption of equal variances was violated. Initial power analyses indicated that a minimum of 159 participants were needed to detect moderate effects (with α=0.05 and 80% power). Statistical analyses were conducted using SPSS Statistics (version 29; IBM Corp).

Future analyses will center on longitudinal changes in PROMs and PREMs, using repeated measures and mixed model analyses. The role of mediating and moderating variables (eg, socioeconomic status, comorbidity, and recovery strength) will be assessed, along with differences between treatment modalities. All statistical analyses will be conducted using R studio (Posit PBC) and SPSS Statistics (IBM Corp).

## Results

In total, 189 individuals participated in the OMER-BE study at baseline, of which 161 (85.2%) participants underwent residential treatment (81 participants treated in a SUD treatment ward in a psychiatric facility and 80 participants in a drug-free TC). Additionally, 28 (14.8%) participants were recruited in outpatient services. At the 45-day follow-up assessment, 120 participants completed the questionnaire, resulting in an overall attrition rate of 36.5% (69/189). The proportion of participants who did not complete the follow-up at 45 days was 38.8% (31/80) in drug-free TCs, 34.6% (28/81) in SUD treatment wards within psychiatric facilities, and 35.7% (10/28) in outpatient services.

Details regarding the baseline sociodemographic and clinical characteristics of the participant sample are presented in Table 1. The average age is 35.5 (SD 9.9) years. The majority of participants were male (n=156, 82.5%), completed secondary education as their highest level of education (n=114, 60.3%), and lived alone (n=90, 47.6%). Most participants were born in Belgium (n=178, 94.2%) and resided in psychiatric facilities (n=81, 42.9%) and TCs (n=80, 42.3%) during the baseline assessment. A total of 153 (81%) participants received previous treatment for SUDs. The most frequently reported main substances were alcohol (n=100, 53.8%), cocaine (n=81, 43.5%), and cannabis (n=64, 34.4%), indicating the presence of many problematic poly-substance users.

**Table 1:** Overview of the sociodemographic and clinical characteristics of the participant sample.

Variables		Total (n=189)	PC^a^ (n=81)	TC^b^ (n=80)	Outpatient (n=28)	*P* value	
Age (years), mean (SD)^c^		35.5 (9.9)	36.6 (11.7)	34.1 (7.9)	36.5 (9.3)	.20^d^	
Sex (male), n (%)		156 (82.5)	63 (77.8)	71 (88.8)	22 (78.6)	.16	
**Education level, n (%)**							.04
	Primary	45 (23.8)	14 (17.3)	23 (28.7)	8 (28.6)		
	Secondary	114 (60.3)	47 (58)	51 (63.7)	16 (57.1)		
	Higher	30 (15.9)	20 (24.7)	6 (7.5)	4 (14.3)		
**Current living situation, n (%)**							.37^e^
	Alone	90 (47.6)	36 (44.4)	39 (48.8)	15 (53.6%)		
	Alone with children	9 (4.8)	4 (4.9)	5 (6.3)	0 (0)		
	Living together with partner and children	18 (9.5)	8 (9.9)	6 (7.5)	4 (14.3)		
	Living together with partner without children	13 (6.9)	8 (9.9)	2 (2.5)	3 (10.7)		
	Living together with others	59 (31.2)	25 (30.9)	28 (35)	6 (21.4)		
**Country of birth, n (%)**							
	Belgium	178 (94.2)	74 (91.4)	76 (95)	28 (100)	.27^e^	
	Belgium (father)	156 (82.5)	64 (79)	65 (81.3)	27 (96.4)	.10	
	Belgium (mother)	161 (85.2)	68 (84)	66 (82.5)	27 (96.4)	.19	
Previous treatment for SUDs^f^ (yes), n (%)		153 (81)	67 (82.7)	68 (85)	18 (64.3)	.048	
Opioid agonist therapy		37 (19.6)	9 (11.1)	15 (18.8)	13 (46.4)	<.001	
**Main substances (n=186)^g,h^, n (%)**							
	Alcohol	100 (53.8)	52 (64.2)	38 (48.7)	10 (37)	.03	
	Amphetamines	41 (22)	9 (11.1)	23 (29.5)	9 (33.3)	.006	
	Benzodiazepines	20 (10.8)	8 (9.9)	12 (15.4)	0 (0)	.08	
	Cannabis	64 (34.4)	23 (28.4)	31 (39.7)	10 (37)	.31	
	Crack	33 (17.7)	9 (11.1)	19 (24.4)	5 (18.5)	.09	
	Codeine + Promethazine	2 (1.1)	2 (2.5)	0 (0)	0 (0)	.63^e^	
	Cocaine	81 (43.5)	21 (25.9)	50 (64.1)	10 (37)	<.001	
	GHB^i^	14 (7.5)	1 (1.2)	10 (12.8)	3 (11.1)	.02	
	Hallucinogens	3 (1.6)	0 (0)	2 (2.6)	1 (3.7)	.21^e^	
	Ketamine	20 (10.8)	13 (16)	5 (6.4)	2 (7.4)	.12	
	New psychoactive substances	6 (3.2)	2 (2.5)	3 (3.8)	1 (3.7)	.87^e^	
	Opioids	35 (18.8)	10 (12.3)	14 (17.9)	11 (40.7)	.005	
Number of main substances (n=186)^c,g,h^, mean (SD)		2.3 (1.4)	1.9 (1.3)	2.7 (1.4)	2.3 (1.4)	.001	
More than one main substance (n=186)^g,h^, n (%)		111 (59.7)	33 (40.7)	60 (76.9)	18 (66.7)	<.001	

^a^PC: psychiatric treatment center.

^b^TC: therapeutic community.

^c^ANOVA.

^d^Welch test.

^e^Fisher exact test.

^f^SUD: substance use disorder.

^g^Some service users reported more than one main substance used.

^h^Data missing for three participants.

^i^GHB: gamma-hydroxybutyric acid.

Initial comparisons were made between the three treatment modalities (Table 1). When considering sociodemographic and clinical characteristics, no significant differences were found in terms of age, sex, living situation, and country of birth (all *P*>.05). However, significant differences were observed regarding education level, history of SUD treatment, OAT, and the primary substances reported. Post hoc analyses revealed that participants in the PC group had the highest level of education, followed by those in the outpatient group, and finally, the individuals from the TC group. On average, 83% (67/81) of participants from the PC group and 85% (68/80) of the participants from the TC group had a previous history of SUD treatment, with no statistically significant difference between the two groups. Moreover, a significantly higher percentage of participants in the outpatient group (13/28, 46%) were engaged in some form of OAT (*P*<.001). This was followed by participants in the TC group (15/80, 19%), and finally, the PC group (9/81, 11%). In terms of substance use, alcohol was more frequently reported as the primary substance in the PC group, followed by TC and outpatient groups. In contrast, opioids were most frequently reported in the outpatient group, followed by TC and PC groups. Amphetamine (*P*=.006), cocaine (*P*<.001), and gamma-hydroxybutyric acid (*P*=.02) were significantly more reported in the TC group, followed by the outpatient, and finally, the PC group. A significantly higher percentage of participants in the TC group reported more than one primary substance, followed by the outpatient and PC group (*P*<.001).

Further comparisons were made regarding comorbid psychiatric conditions and PROMs at baseline (Table 2). No significant differences were found in levels of PTSD, depression, anxiety, and stress scores between the treatment modalities (all *P*>.05). However, significant differences were observed in scores on the ADHD Self-Report scale, with participants in the outpatient group scoring significantly lower compared to the TC group (*P*=.03). No significant differences were found in general health and quality of life scores (all *P*>.05). In contrast, the total score on the SURE questionnaire revealed significant differences at baseline between the groups, with participants in the outpatient group scoring lower overall than those in the other groups (*P*<.001). This trend was significant in the subdomains of “Substance Use” (*P*<.001), “Self-care” (*P*<.001), and “Outlook on Life” (*P*=.008).

**Table 2:** Comparison of comorbidity and PROMs^a^ at baseline.

Questionnaires		Total (n=189), mean (SD)	PC^b^ (n=81), mean (SD)	TC^c^ (n=80), mean (SD)	Outpatient (n=28), mean (SD)	*P* value
PC-PTSD-5^d^		2.24 (2.00)	2.09 (1.92)	2.51 (2.07)	1.89 (1.95)	.25
**DASS 21^e^**						
	Depression	18.94 (10.92)	17.58 (10.80)	20.23 (10.80)	19.21 (11.54)	.31
	Anxiety	14.03 (9.50)	12.94 (9.12)	15.53 (9.96)	12.93 (8.92)	.18
	Stress	19.31 (10.15)	18.00 (10.47)	21.00 (9.72)	18.29 (10.09)	.15
ASRS^f^		3.44 (1.68)	3.36 (1.73)	3.75 (1.50)	2.79 (1.89)	.03
**PROMIS GH^g^**						
	Physical	2.77 (.86)	2.73 (.92)	2.85 (.83)	2.77 (.86)	.55
	Mental	2.59 (.92)	2.62 (.94)	2.61 (.88)	2.46 (.96)	.73
**SURE^h^ total**		52.05 (7.70)	52.19 (7.99)	54.03 (5.46)	46.00 (9.31)	<.001^i^
	Substance use	14.97 (2.89)	15.07 (2.98)	15.60 (2.42)	12.89 (3.01)	<.001
	Self-care	12.02 (2.78)	11.81 (3.00)	12.91 (1.97)	10.07 (3.07)	<.001^i^
	Relationships	10.90 (1.61)	10.98 (1.60)	11.13 (1.26)	10.04 (2.20)	.05^i^
	Material resources	7.56 (1.76)	7.72 (1.73)	7.48 (1.80)	7.36 (1.77)	.55
	Outlook on life	6.59 (1.88)	6.60 (1.92)	6.91 (1.66)	5.64 (2.08)	.008
**WHOQoL-BREF^j^**						
	Perception quality of life	2.94 (.82)	2.93 (.77)	2.95 (.87)	2.96 (.84)	.97
	Perception health	2.85 (.96)	2.91 (.91)	2.81 (.98)	2.79 (1.03)	.74
	Physical health	13.52 (2.63)	13.33 (2.77)	13.85 (2.41)	13.14 (2.80)	.33
	Psychological health	11.43 (2.81)	11.68 (2.67)	11.33 (2.88)	10.98 (3.01)	.48
	Social relationships	12.14 (3.70)	12.59 (3.72)	11.65 (3.55)	12.24 (3.98)	.27
	Environment	13.24 (2.87)	13.74 (2.84)	12.98 (2.85)	12.55 (2.84)	.09

^a^PROM: patient-reported outcome measure.

^b^PC: psychiatric treatment center.

^c^TC: therapeutic community.

^d^PC-PTSD-5: Primary Care Posttraumatic Stress Disorder Screen.

^e^DASS 21: Depression, Anxiety, and Stress Scale.

^f^ASRS: Adult Attention-Deficit/Hyperactivity Disorder-Self-Report Scale.

^g^PROMIS GH: Patient-Reported Outcomes Measurement Information System Global Health.

^h^SURE: Substance Use Recovery Evaluator.

^i^Welch test.

^j^WHOQoL-BREF: brief version of the World Health Organization Quality of Life questionnaire.

Additionally, comparisons were conducted of baseline sociodemographic, clinical, and PROM scores between participants who completed the 45-day follow-up and those who did not participate in the 45-day assessment (Table 3). Participants who completed the 45-day follow-up were significantly older (mean age 37.1, SD 9.4 years) compared to noncompleters (mean age 32.8, SD 10.3 years; *P*=.005). In general, noncompleters had a lower level of education (*P*=.009). Significant differences were also found regarding living situations, with a higher proportion of completers living alone compared to noncompleters (*P*=.03). The country of birth of participants’ mothers differed significantly between groups, as a larger proportion of completers had a mother born in Belgium (109/120, 91%) compared to noncompleters (52/69, 75%; *P*=.004). A similar trend was observed for paternal country of birth, but the difference was not statistically significant (*P*=.44). Regarding substance use characteristics, alcohol was more frequently reported as the primary substance among completers (72/120, 61%) compared to noncompleters (28/69, 42%; *P*=.01). However, no significant differences were observed between groups concerning other primary substances of use or the number of primary substances reported (all *P*>.05).

**Table 3:** Comparison of sociodemographic and clinical characteristics of 45-day follow-up completers and noncompleters.

Variables			Total (n=189)		45-day completers (n=120)				Noncompleters (n=69)		*P* value	
Age (years), mean (SD)^a^			35.5 (9.9)		37.1 (9.4)				32.8 (10.3)		.005	
Sex (male), n (%)			156 (82.5)		100 (83.3)				56 (81.2)		.71	
**Education level, n (%)**												.009
	Primary	45 (23.8)		20 (16.7)				25 (36.2)				
	Secondary	114 (60.3)		78 (65)				36 (52.2)				
	Higher	30 (15.9)		22 (18.3)				8 (11.6)				
**Current living situation, n (%)**												.03^b^
	Alone	90 (47.6)		67 (55.8)				23 (33.3)				
	Alone with children	9 (4.8)		6 (5)				3 (4.3)				
	Living together with partner and children	18 (9.5)		11 (9.2)				7 (10.1)				
	Living together with partner without children	13 (6.9)		7 (5.8)				6 (8.7)				
	Living together with others	59 (31.2)		29 (24.2)				30 (43.5)				
**Country of birth, n (%)**												
	Belgium	178 (94.2)		113 (94.2)				65 (94.2)		.99^b^		
	Belgium (father)	156 (82.5)				101 (84.2)		55 (79.7)		.44		
	Belgium (mother)	161 (85.2)				109 (90.8)		52 (75.4)		.004		
Previous treatment for SUDs^c^ (yes), n (%)			153 (81)				98 (81.7)		55 (79.7)		.74	
Opioid agonist therapy, n (%)			37 (19.6)				19 (15.8)		18 (26.1)		.09	
**Main substances (n=186)^d,e^, n (%)**												
	Alcohol	100 (53.8)				72 (60.5)		28 (41.8)				.01
	Amphetamines	41 (22)				27 (22.7)		14 (20.9)		.78		
	Benzodiazepines	20 (10.8)				15 (12.6)		5 (7.5)		.28		
	Cannabis	64 (34.4)				37 (31.1)		27 (40.3)		.21		
	Crack	33 (17.7)				22 (18.5)		11 (16.4)		.72		
	Codeine + Promethazine	2 (1.1)				1 (0.8)		1 (1.5)		.99^b^		
	Cocaine	81 (43.5)				52 (43.7)		29 (43.3)		.96		
	GHB^f^	14 (7.5)				12 (10.1)		2 (3)		.08		
	Hallucinogens	3 (1.6)				3 (2.5)		0 (0)		.55^b^		
	Ketamine	20 (10.8)				9 (7.6)		11 (16.4)		.06		
	New psychoactive substances	6 (3.2)				2 (1.7)		4 (6)		.19^b^		
	Opioids	35 (18.8)				19 (16)		16 (23.9)		.19		
Number of main substances (n=186)^a,d,e^, mean (SD)			2.3 (1.4)				2.3 (1.3)		2.2 (1.5)		.75	
More than one main substance (n=186)^d,e^, n (%)			111 (59.7)				75 (63)		36 (53.7)		.22	
**Treatment modality, n (%)**												.86
	TC^g^	80 (42.3)				49 (40.8)		31 (44.9)				
	PC^h^	81 (42.9)				53 (44.2)		28 (40.6)				
	Outpatient	28 (14.8)				18 (15)		10 (14.5)				

^a^ANOVA.

^b^Fisher exact test.

^c^SUD: substance use disorder.

^d^Some service users reported more than one main substance used.

^e^Data missing for three participants.

^f^GHB: gamma-hydroxybutyric acid.

^g^TC: therapeutic community.

^h^PC: psychiatric treatment center.

In terms of PROMs, a significant difference was observed in the SURE domain “material resources,” where completers had significantly higher scores (mean 7.78, SD 1.62) than noncompleters (mean 7.19, SD 1.94; *P*=.04; Table 4). Additionally, noncompleters scored significantly higher (mean 2.99, SD 0.87) on the physical health domain of the PROMIS Global Health compared to people who completed the 45-day follow-up (mean 2.65, SD 0.84; *P*=.01), indicating better perceived physical health. No significant differences were found between completers and noncompleters across other PROM domains (all *P*>.05).

**Table 4:** Comparison of comorbidity and PROMs^a^ at baseline between 45-day follow-up completers and noncompleters.

Questionnaires		Total (n=189), mean (SD)			45-day completers (n=120), mean (SD)		Noncompleters (n=69), mean (SD)		*P* value
PC-PTSD-5^b^		2.24 (2.00)			2.30 (2.00)		2.13 (2.00)		.58
**DASS 21^c^**									
	Depression	18.94 (10.92)			18.43 (10.77)		19.83 (11.21)		.40
	Anxiety	14.03 (9.50)			14.30 (9.66)		13.57 (9.25)		.61
	Stress	19.31 (10.15)			18.70 (10.38)		20.38 (9.72)		.28
ASRS^d^		3.44 (1.68)			3.35 (1.74)		3.59 (1.58)		.34
**PROMIS GH^e^**									
	Physical	2.77 (.86)			2.65 (.84)		2.99 (.87)		.01
	Mental	2.59 (.92)			2.56 (.87)		2.65 (1.00)		.49
**SURE^f^ total**		52.05 (7.70)			52.73 (7.33)		50.87 (8.22)		.11
	Substance use	14.97 (2.89)			15.21 (2.75)		14.57 (3.10)		.14
	Self-care	12.02 (2.78)			12.15 (2.71)		11.80 (2.90)		.40
	Relationships	10.90 (1.61)			10.99 (1.52)		10.74 (1.75)		.29
	Material resources	7.56 (1.76)			7.78 (1.62)		7.19 (1.94)		.04^g^
	Outlook on life	6.59 (1.88)			6.60 (1.84)		6.58 (1.94)		.94
**WHOQoL-BREF^h^**									
	Perception quality of life		2.94 (.82)	2.94 (.80)		2.94 (.86)		.99	
	Perception health		2.85 (.96)	2.83 (.96)		2.88 (.95)		.73	
	Physical health		13.52 (2.63)	13.51 (2.70)		13.55 (2.52)		.92	
	Psychological health		11.43 (2.81)	11.26 (2.68)		11.72 (3.01)		.28	
	Social relationships		12.14 (3.70)	11.83 (3.83)		12.68 (3.40)		.13	
	Environment	13.24 (2.87)			13.40 (2.87)		12.96 (2.86)		.30

^a^PROM: patient-reported outcome measure.

^b^PC-PTSD-5: Primary Care Posttraumatic Stress Disorder Screen.

^c^DASS 21: Depression, Anxiety, and Stress Scale.

^d^ASRS: Adult Attention-Deficit/Hyperactivity Disorder Self-Report Scale.

^e^PROMIS GH: Patient-Reported Outcomes Measurement Information System Global Health.

^f^SURE: Substance Use Recovery Evaluator.

^g^Welch test.

^h^WHOQoL-BREF: brief version of the World Health Organization Quality of Life questionnaire.

## Discussion

### Principal Findings

The use of PROMs and PREMs in SUD treatment services is limited to date, and no systematic monitoring system of patient-reported outcomes in Belgium is currently available [34]. Therefore, the OMER-BE study was set up to assess PROMs and PREMs systematically and to improve the quality of SUD services through the routine measurement and monitoring of patient-reported outcomes and experiences at regular times during and after treatment.

Comparisons across the three treatment modalities at baseline revealed several noteworthy differences and similarities. While no significant differences were observed in age, sex, living situation, or country of birth, significant variations in education levels, history of SUD treatment, engagement in OAT, and primary substance of problematic use were observed. Regarding co-occurring mental health disorders, no significant differences were found in PTSD, depression, anxiety, and stress scores between the treatment modalities. However, significant differences were noted in ADHD scores, with the TC group scoring significantly higher than the outpatient group. These differences can be attributed to several factors. TCs are often more suitable for individuals with more severe ADHD symptoms, as these environments provide the structured support and comprehensive care needed to manage both SUDs and comorbid conditions. These settings allow for intensive monitoring and support, which may be crucial for individuals struggling with ADHD symptoms, such as impulsivity [55].

Analysis of PROM scores showed no significant differences between treatment modalities in general health and quality of life. However, significant differences were found in recovery strengths, with participants in the outpatient group scoring lower overall than those in the other groups. These differences, particularly noted in the subdomains of “Substance Use,” “Self-care,” and “Outlook on Life,” suggest that the structure of the treatment environment plays a role in shaping recovery trajectories. For example, we observed that participants in residential treatment score significantly higher on the “Substance Use” subscale compared to those in outpatient treatment, suggesting lower levels of substance use among those in residential settings. This difference can be attributed to the structured and controlled environment of residential treatment settings, where strict measures are often in place to encourage abstinence and limit access to substances [56]. The supervision and supportive community in residential facilities may also play a crucial role in reducing substance use, which is less enforceable in outpatient settings where individuals have more autonomy and access to substances. The significant difference in the “Self-care” subscale, with residential participants scoring higher, may reflect the comprehensive care and support provided in residential settings. These environments typically offer structured daily routines and more intensive psychological and physical care [56]. In contrast, outpatient treatment often places a greater emphasis on self-management [57], which can be challenging for individuals with limited resources or support networks. Participants in residential treatment also scored significantly higher on the “Outlook on Life” subscale, which may be attributed to the supportive environment of residential treatment settings, where participants are provided with continuous care and peer support, all of which contribute to a more positive and optimistic outlook on life. Notably, the group difference in the “Relationship” subscale was close to significance. As participants were assessed in the first weeks of treatment, it is possible that participants in residential facilities did not have enough time to build meaningful relationships with other service users and providers.

In addition to differences between treatment modalities, comparisons between participants who completed the 45-day follow-up and those who did not participate in this assessment revealed several significant differences. Participants who completed the 45-day follow-up were significantly older, had a higher education level, were more likely to live alone, and were more likely to have a mother born in Belgium. Additionally, they scored higher on the “Material Resources” domain of the SURE, which includes questions about stable housing, a steady income, and effective financial management. These findings are in line with studies that suggest that factors such as lower education level, younger age, unemployment, and financial instability are associated with higher attrition in follow-up assessments [58,59]. Furthermore, some studies have found that factors such as low socioeconomic status are associated with limitations in people’s ability to use electronic devices [21,60,61], which may contribute to reduced participation in follow-up assessments. The higher completion rates among individuals living alone could indicate that they experience fewer external obligations that interfere with follow-up participation. The differences observed in maternal country of birth suggest that cultural or socioeconomic factors may also play a role in study retention, such as language barriers or differing levels of trust in research participation. Furthermore, alcohol was more frequently reported as the primary substance of use among completers and individuals with alcohol as primary substance of use had a lower attrition rate in comparison to the total sample (28% vs 36.6%). The higher prevalence of alcohol as a primary substance among completers may reflect differences in treatment pathways and more stable living situations that can be linked to socioeconomic factors and higher education levels.

### Limitations

Despite being innovative and pioneering, the proposed study also has some limitations. First, recruiting participants in outpatient facilities proved to be extremely challenging, leading to a limited number of participants in these settings. One significant obstacle was the presence of long waiting lists, resulting in a limited number of new treatment episodes and lower turnover rates compared to the residential services. The frequency of contact between potential participants and service providers was limited in many outpatient settings, as many service users have only weekly or biweekly appointments. This poses significant scheduling constraints, especially since participants were expected to complete the baseline questionnaires within three weeks of treatment initiation. Additionally, there was a high occurrence of no-shows for scheduled appointments with the researchers, which resulted in missed opportunities to engage potential participants. To address these challenges, several strategies were implemented. Flyers and posters were provided in waiting areas to inform potential participants about the study and proactive follow-up calls were made to confirm appointments. Moreover, regular contact was maintained with service providers (both through email and phone) to encourage participation. Despite these efforts, maintaining consistent engagement in outpatient facilities has been proven particularly challenging, resulting in some outpatient centers withdrawing from the study. Consequently, future comparative analyses will primarily focus on residential treatment services due to the limited number of outpatient participants. These challenges raise important considerations for the broader implementation of PROMs and PREMs in outpatient addiction services. While comprehensive assessments seem feasible in residential settings, these findings underscore the need for effective strategies for the structural implementation of PROMs and PREMs in outpatient services. Potential strategies to increase participation and retention may include broadening recruitment periods, simplifying assessment batteries, and using short-form PROMs and PREMs that could be completed during brief waiting periods before appointments. Additionally, codeveloping implementation strategies with service users and providers could further enhance feasibility and engagement in outpatient settings.

Second, recruiting facilities and participants in the French-speaking regions of Belgium proved to be more challenging than in the Dutch-speaking parts. This might be caused by differing attitudes toward the importance of outcome evaluation and measurement. Hence, beyond providing feasible measurement instruments and implementation strategies, there is a need to build service cultures that value the implementation of measurements to improve the quality of support. Organizing policies and funding that support the routine use of PROMs and PREMs might be an important step toward realizing this. Yet, this also includes overcoming practical challenges such as staffing shortages that hinder broader participation.

Finally, the study is limited by the lack of standardized diagnostic assessment of participants entering the study. Inclusion was based on proxy indicators (previous treatment for SUD, clinical assessment, or history of substance misuse), rather than on clinical diagnoses. This lack of structural diagnosis may have introduced variability in our findings and may limit generalization to other populations.

### Conclusions

Despite these challenges, this study is one of the first studies to explore the assessment of PROMs and PREMs in SUD treatment services using the ICHOM SSA set of instruments. By systematically and routinely monitoring PROMs and PREMs, the project aims to empower service providers and give them tools to evaluate subjective treatment outcomes and experiences. This approach can provide service providers and policy makers with benchmarks for assessing outcomes and experiences during and after treatment across various treatment modalities. By applying an internationally validated tool, the study allows international comparison with similar interventions and treatment modalities globally. Future analyses will explore longitudinal changes in PROMs and PREMs, with a particular focus on the influence of baseline mediating and moderating variables (eg, socioeconomic status, comorbidity, and recovery strength) and differences between treatment modalities. The findings from this study will offer new perspectives and insights into the effectiveness of different SUD treatment modalities and their impact on diverse service user populations. This shift toward person-centered care not only supports better recovery outcomes but also fosters continuous improvement and innovation in SUD treatment services. Ultimately, systematically monitoring PROMs and PREMs has the potential to enhance the quality of care by offering a comprehensive understanding of service users’ needs and treatment effectiveness, extending beyond abstinence.

## Abbreviations

ADHD: attention-deficit/hyperactivity disorder
ICHOM: International Consortium for Health Outcomes Measurement
OAT: opioid agonist therapy
OMER-BE: Outcome Measurement and Evaluation as a Routine Practice in Alcohol and Other Drug Services in Belgium
PC: psychiatric treatment center
PREM: patient-reported experience measure
PREMAT: Patient-Reported Experience Measure for Addiction Treatment
PROM: patient-reported outcome measure
PROMIS: Patient-Reported Outcomes Measurement Information System
PTSD: posttraumatic stress disorder
SSA: Standard Set for Addictions
SUD: substance use disorder
SURE: Substance Use Recovery Evaluator
TC: therapeutic community
WHOQoL-BREF: brief version of the World Health Organization Quality of Life questionnaire

## Data Availability

The datasets generated and analyzed during this study are available from the corresponding author upon reasonable request.
